# Potential role of SIRT1 in cell ferroptosis

**DOI:** 10.3389/fcell.2025.1525294

**Published:** 2025-03-05

**Authors:** Yueming Zhang, Fanxiao Kong, Nan Li, Lina Tao, Jinghui Zhai, Jie Ma, Sixi Zhang

**Affiliations:** ^1^ Department of Clinical Pharmacy, The First Hospital of Jilin University, Jilin, China; ^2^ State Key Laboratory of Bioactive Substance and Function of Natural Medicines, Institute of Materia Medica, Chinese Academy of Medical Sciences and Peking Union Medical College, Beijing, China; ^3^ Department of Pharmacy, The First Hospital of Jilin University, Jilin, China

**Keywords:** SIRT1, ferroptosis, molecular mechanisms, therapeutic strategies, various diseases

## Abstract

Ferroptosis is a novel form of cell death that uniquely requires iron and is characterized by iron accumulation, the generation of free radicals leading to oxidative stress, and the formation of lipid peroxides, which distinguish it from other forms of cell death. The regulation of ferroptosis is extremely complex and is closely associated with a spectrum of diseases. Sirtuin 1 (SIRT1), a NAD + -dependent histone deacetylase, has emerged as a pivotal epigenetic regulator with the potential to regulate ferroptosis through a wide array of genes intricately associated with lipid metabolism, iron homeostasis, glutathione biosynthesis, and redox homeostasis. This review provides a comprehensive overview of the specific mechanisms by which SIRT1 regulates ferroptosis and explores its potential therapeutic value in the context of multiple disease pathologies, highlighting the significance of SIRT1-mediated ferroptosis in treatment strategies.

## 1 Introduction

Ferroptosis, first identified in 2012, is a unique form of regulated cell death that is distinct from traditional apoptosis, necroptosis, and senescence (Dixon et al., 2012). Cells undergoing ferroptosis typically display shrunken mitochondria, increased mitochondrial membrane density, loss of mitochondrial cristae, reduced mitochondrial membrane potential, and rupture of the outer mitochondrial membrane (Li et al., 2024a; Shan et al., 2024; Lin et al., 2024a). The occurrence of ferroptosis is intricately linked to several critical biochemical processes, including the disruptions in iron homeostasis, limited synthesis of glutathione (GSH), and the accumulation of lipid peroxides (Tian et al., 2024; Cai et al., 2024; Li et al., 2024b; Yang et al., 2024). Excess iron is typically stored in ferritin to prevent it from catalyzing the formation of hydroxyl radicals, which can react with polyunsaturated fatty acids in the cell and plasma membranes. This reaction leads to the generation of a significant amount of lipid reactive oxygen species (ROS), contributing to the cellular demise that is characteristic of ferroptosis. The system XC is pivotal in sustaining cellular GSH levels by mediating the cellular uptake of cystine. GSH acts as a cofactor for antioxidant enzymes such as GPX4, which play a role in the elimination of ROS. These pathways form an interconnected network that safeguards cells against ferroptosis by maintaining a delicate balance between antioxidant defense and the production of reactive species. Ferroptosis s closely related to the occurrence and development of a variety of diseases, such as nervous system diseases, heart diseases, liver diseases, gastrointestinal diseases, lung diseases, kidney diseases, pancreatic diseases tumors, kidney injury, tumor, etc. By targeting the key components of ferroptosis, disease progression can be slowed, offering promising treatment strategies for many diseases.

Sirtuins (SIRTs), a subset of NAD + -dependent histone deacetylases, are evolutionarily conserved and consist of seven isoforms. Among these, SIRT1-3 have been implicated in the regulation of ferroptosis, with SIRT1 being the most extensively studied. SIRT1 is widely expressed across tissues and organs such as the brain, heart, liver, kidneys, and skeletal muscle, with particularly high expression in tissues vulnerable to oxidative stress and those with high metabolic activity. SIRT1 exhibits diverse subcellular localizations, being found in the nucleus, cytoplasm, or both, depending on the cell type (Sgadari et al., 2023). Structurally, it is composed of 747 amino acid (aa) residues, with both C- and N-terminal domains contributing to its structure and function. The C-terminal domain, comprising 25 aa residues, is crucial for SIRT1’s catalytic activity. It forms a hairpin that interacts with the β-sheet of the NAD + -binding domain, while the N-terminal domain enhances the enzyme’s activity (Davenport et al., 2014). As a central modulator of ferroptosis, SIRT1 modulates key ferroptosis-related proteins through deacetylation, thereby enhancing cellular resilience against ferroptotic cell death. Its regulatory role has significant implications for the treatment and management of diseases associated with oxidative stress and ferroptosis. Recent studies have highlighted SIRT1’s potential in inhibiting ferroptosis by influencing pathways related to glutathione synthesis, antioxidant mechanisms, and the metabolism of lipids and iron. Despite the growing evidence of SIRT1’s involvement in ferroptosis, a comprehensive understanding of the regulatory interplay between SIRT1 and ferroptosis signaling pathways is yet to be fully elucidated (Dang et al., 2022; Wang et al., 2021; Qiongyue et al., 2022). In this review, we delve into the current evidence of the functions of SIRT1 in regulating ferroptosis and therapeutic potential in various diseases. We aim to consolidate current understanding and explore the therapeutic implications of targeting SIRT1-mediated ferroptosis, offering insights into its promise as an innovative pathway for developing treatment strategies.

## 2 Regulation of SIRT1 on ferroptosis

### 2.1 Redox homeostasis

Reactive oxygen species (ROS) are central to the process of ferroptosis, as they facilitate lipid peroxidation and damage cell membranes. Therefore, regulating ROS levels may be an important strategy for controlling ferroptosis and related diseases. The System XC and GPX4 are vital in maintaining intracellular GSH levels, which are essential for ROS removal. Notably, SIRT1 functions as a sensor of redox changes and plays a critical protective role against ferroptosis by regulating redox homeostasis via Nuclear factor erythroid 2-related factor 2 (Nrf2) and p53 (Liang et al., 2023).

#### 2.1.1 Role of SIRT1- Nrf2 in ferroptosis

Nuclear factor erythroid 2-related factor 2 (Nrf2) is a key transcription factor that maintains redox balance and protects cells from oxidative damage. Accumulating evidence points that Nrf2 upregulation can suppress the initiation of ferroptosis (Bellezza et al., 2012; Sun et al., 2016; Wang et al., 2023a; Lv et al., 2021). SIRT1 modulates various components within the antioxidant system, such as Heme Oxygenase-1(HO-1), glutathione (GSH), catalase (CAT), superoxide dismutase 1(SOD1) and SOD2 through deacetylation of Nrf2 (Zhang et al., 2018; Abukhalil et al., 2025; Xia et al., 2023). Recent studies have illustrated the role of the SIRT1-Nrf2-HO-1 pathway in the regulation of ferroptosis. Wang *et al.* have shown that SIRT1-Nrf2-HO-1 activation attenuated lipid peroxide accumulation and inhibited ferroptosis (Wang et al., 2023a). Another study demonstrated that SIRT1 activation positively regulates the Nrf2/HO-1 pathway, reducing mitochondrial damage and ferroptosis. Furthermore, HO-1 may play a role in modulating GPX4 levels (Xie et al., 2022). Dang et al. pointed that SIRT1 activation may mediate the upregulation of GPX4 levels by Nrf2-HO-1 axis in the alleviation of neuronal injury. Significant strides have been made in recent years to elucidate the protective effects of SIRT1-Nrf2 activation against ferroptosis (Xia et al., 2023; Xie et al., 2022), and further exploration of the underlying regulatory mechanisms is warranted.

#### 2.1.2 Role of SIRT1-p53 in ferroptosis

p53 is a multifunctional protein that plays a crucial role in regulating intracellular levels of reactive oxygen species (ROS) and modulating ferroptosis through targeting downstream molecules (Latunde-Dada, 2017). The complex interplay between SIRT1 and p53 is crucial in managing ferroptosis activation, primarily through inhibition of p53’s pro-ferroptotic activity and promotion of p21 and GSH synthesis. First, overexpression of SIRT1 has been shown to repress p53 transcriptional activity, increasing SLC7A11 levels and inhibiting ferroptosis (Ma et al., 2020). Zhao et al. reported that SIRT1 participates in the development of gastric cancer by targeting p53 to regulate ferroptosis (Zhao et al., 2023a). In gastric cancer cells, silencing SIRT1 leads to upregulation of p53 and downregulation of SLC7A11, indicating that SIRT1 suppression promotes ferroptosis. Second, p21, a downstream target of p53, can form a complex with p53 and influence its transcriptional activity. SIRT1 enhances p21 expression by modulating p53 activity, which may contribute to cellular redox balance and ferroptosis resistance by mitigating oxidative stress. Through this pathway, SIRT1 indirectly supports antioxidant defenses, including GSH synthesis, further reducing ferroptosis susceptibility (Gu et al., 2022; Wang et al., 2025). While these findings highlight SIRT1’s protective role in ferroptosis regulation via the p53/p21 axis, further research is needed to elucidate its context-specific effects across different disease models.

### 2.2 Iron homeostasis

Iron homeostasis is crucial for maintaining normal cellular and physiological metabolism. When iron supply is abundant, iron storage protein ferritin synthesis increases to store the excess. Iron (Fe^2+^) can be oxidized to Fe^3+^ by the ferroxidase hephaestin (Heph) and bind to transferrin (TF) on cell membranes, forming the TF-Fe^3+^ complex, which is facilitated by the presence of apo-Tf. Apo-Tf acts as an iron acceptor molecule, enhancing iron (Fe^2+^) efflux from cells via ferroportin (FPN1), and thus enhancing iron export and absorption (Weichhart, 2024). Most intracellular iron is either found in heme-containing and mitochondrial proteins or stored by ferritin as Fe3^+^, preventing iron overload that could lead to oxidative stress. Ferritin plays a vital role in preserving iron balance by storing and releasing iron. However, excessive iron levels can increase the labile iron pool (LIP), elevate intracellular reactive oxygen species (ROS), and lead to the accumulation of lipid peroxides, ultimately promoting ferroptosis. SIRT1 has been shown to influence ferroptosis by regulating iron metabolism. Activation of the SIRT1/Nrf2 pathway, such as by alpha lipoic acid, can modulate iron metabolism and mitigate ferroptosis by up-regulating ferritin and ferritin heavy chain 1 (FTH1), and down-regulating the iron import protein divalent metal transporter 1 (DMT1) (Zheng et al., 2023). Lv et al. demonstrated that suppression of the SIRT1/Nrf2/HO-1/GPX4 pathway and FTH1 protein can exacerbate ferroptosis, underscoring the significance of the SIRT1-Nrf2 signaling pathway in iron metabolism and ferroptosis (Lv et al., 2024). Additionally, SIRT1 activation is linked to altered hepcidin production and increased ferritinophagy, which can suppress ferroptosis and prevent the detrimental effects of elevated cytosolic iron (Su et al., 2021; Tziastoudi et al., 2023). However, Zhou et al. noted an exception to the inhibitory effect of SIRT1 on ferroptosis, where intestinal SIRT1 deficiency improves iron metabolism in ethanol-induced hepatic injury in mice by ameliorating iron dysfunction and alleviating ferroptosis in hepatocytes. This suggests that the role of SIRT1 can be context-dependent (Zhou et al., 2020). In summary, SIRT1 plays a multifaceted role in regulating iron metabolism and influencing ferroptosis. Modulating SIRT1 can protect against ferroptosis by influencing key iron-related proteins and pathways. However, the specific outcomes of SIRT1’s actions can vary depending on the cellular context and the type of stress involved. Further research is needed to fully understand the complex interactions between SIRT1, iron metabolism, and ferroptosis, which could potentially lead to the development of novel therapeutic strategies for iron-related diseases.

### 2.3 Lipid peroxidation metabolism

**FIGURE 1:**
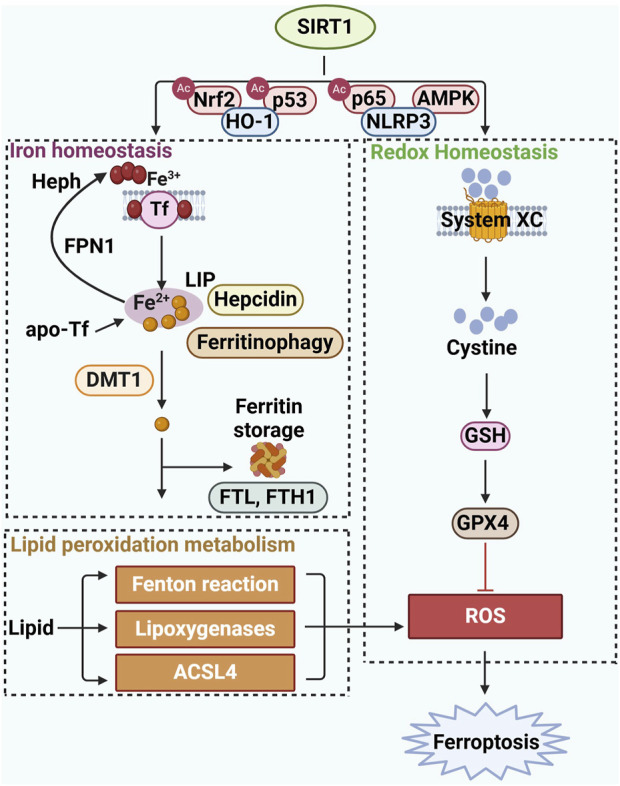
The specific mechanism of SIRT1 in regulating ferroptosis.

Iron can exacerbate the accumulation of lipid peroxides by catalyzing Fenton reaction, which in turn disrupts the intracellular redox balance, leading to an attack on biomolecules and ultimately culminating in ferroptosis. SIRT1, a key metabolic factor in energy regulation, can stimulate various endocrine signals related to lipid metabolism. and an increasing number of studies have shown that SIRT1 is involved in endocrine and metabolic diseases (Lu et al., 2023). The long-chain fatty acyl CoA synthetase (ACSLs) family of enzymes, which significantly contributes to lipid metabolism, has been recognized as a crucial regulator in the process of ferroptosis (Dixon et al., 2015). Emerging research suggests that SIRT1 activation may reduce ACSL4 expression levels, potentially alleviating the effects of ferroptosis (Majeed et al., 2021; Wang et al., 2020; Yu et al., 2023a). The work by Chen et al. provides compelling evidence that overexpression of SIRT1 can inhibit lipid peroxidation and decrease malondialdehyde (MDA) levels, a marker of lipid peroxidation. Furthermore, SIRT1 overexpression can reverse the typical upregulation of ACSL4 and acetylated p53, and the downregulation of SLC7A11 and GPX4 observed in ferroptosis, thus inhibiting ferroptotic cell death (Chen et al., 2022a). These findings underscore SIRT1’s potential in modulating lipid metabolism and its protective role against ferroptosis. However, the precise mechanisms by which SIRT1 interacts with ACSL4 to regulate ferroptosis remain to be fully understood, indicating a need for further research in this area. This research could pave the way for developing novel therapeutic strategies that target the SIRT1-ACSL4 axis to combat diseases associated with ferroptosis (Figure 1).

### 2.4 Inflammation

Inflammation and ferroptosis are closely interconnected, with inflammation often promoting ferroptosis through the release of pro-inflammatory cytokines and the induction of oxidative stress. Nuclear Factor kappa B (NF-κB), a key transcription factor, is involved in the regulation of both inflammation and oxidative stress. Upon activation, NF-κB translocates to the nucleus, where it induces the expression of pro-inflammatory and pro-ferroptotic genes, promote iron accumulation and reactive ROS production, leading to lipid peroxidation and ferroptosis (Chen et al., 2024a). By deacetylating the p65 subunit of NF-κB at lysine 310, SIRT1 inhibits its nuclear translocation and transcriptional activity, thereby reducing inflammation, decreasing ROS production and lipid peroxidation, ultimately inhibiting ferroptosis and alleviating cell injury (Min et al., 2025). Conversely, the downregulation of SIRT1 has been shown to activate the NLRP3 inflammasome, leading to the release of pro-inflammatory cytokines like IL-1β and the subsequent disruption of iron homeostasis. This disruption is characterized by increased lipid peroxidation, and the depletion of key antioxidants such as GPX4 and GSH, further weakening the cellular defense against oxidative stress and rendering cells more susceptible to ferroptosis (Hacioglu, 2024). Additionally, under pathological conditions, SIRT1 activation by adenosine 5′-monophosphate (AMP)-activated protein kinase (AMPK), a key energy sensor that regulates cellular metabolism and stress responses, further attenuates inflammatory signaling. Activation of the AMPK/SIRT1 signaling pathway alleviates the degradation of GSH, thus inhibiting ferroptosis. Selective inhibition of SIRT1 weakens the protective effect of the AMPK/SIRT1 signaling pathway against endoplasmic reticulum stress and ferroptosis (Xu et al., 2023). Restoring the activity of AMPK and SIRT1 can effectively inhibit ferroptosis, providing a potential therapeutic strategy for treating related diseases. Interestingly, SIRT1 inhibition subsequently affects the phosphorylation of AMPK, leading to downstream activation of acetyl-CoA carboxylase, promotes the synthesis of polyunsaturated fatty acids, which serve as substrates for lipid peroxidation and ferroptosis induction (Zhang et al., 2023a). Taken together, these findings underscore the critical role of SIRT1 in modulating inflammation-driven ferroptosis through NF-κB, NLRP3 and AMPK signaling. Enhancing SIRT1 activity could serve as a potential therapeutic strategy for mitigating ferroptosis-associated diseases by reducing inflammation, preserving antioxidant defenses, and regulating lipid metabolism. Further studies are warranted to fully elucidate the therapeutic potential of targeting the SIRT-mediated inflammation in ferroptosis-related pathologies.

## 3 SIRT1-mediated ferroptosis and disease

### 3.1 Neuro diseases

**FIGURE 2:**
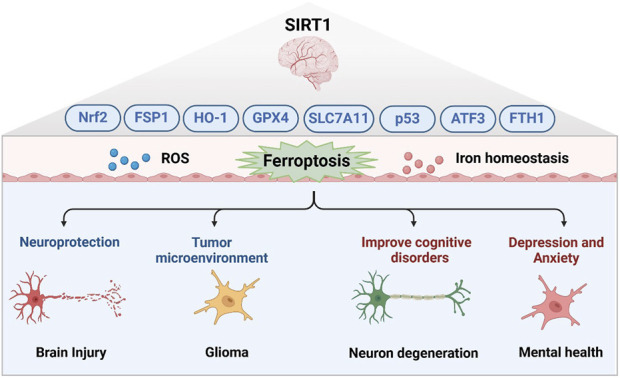
The potential of SIRT1-mediated ferroptosis in neuroprotection and disease treatment.

Studies have delineated the neuroprotective role of SIRT1 in various pathological conditions, including early brain injury following subarachnoid hemorrhage. SIRT1’s role in ferroptosis within brain injury is primarily through the regulation of cellular oxidative stress and iron metabolic balance. By deacetylating a variety of key proteins, SIRT1 enhances the antioxidant capacity of cells, reduces the production of ROS, and inhibits lipid peroxidation, thus alleviating the damage caused by ferroptosis to nerve cells (Hao et al., 2022; Conde et al., 2023; Xie et al., 2022; Liang et al., 2023; Guo et al., 2022). SIRT1 activation also helps to maintain homeostasis of intracellular iron ions, prevent Fenton reaction induced by excess iron ions, and reduce oxidative DNA damage and protein degeneration. Furthermore, SIRT1 inhibits ferroptosis and protects nerve cells from oxidative stress by promoting the expression of antioxidant enzymes like glutathione peroxidase 4 (GPX4) and activating pathways such as Nrf2/HO-1 and ferroptosis suppressor protein 1 (FSP1), playing a vital role in neuroprotection and repair post-brain injury (Liu et al., 2023; Yuan et al., 2022; Chen et al., 2024b; Zhang et al., 2024a). Additionally, SIRT1-mediated ferroptosis is significant in neurodegenerative diseases and cognitive disorders, with activation of the SIRT1/Nrf2 pathway shown to inhibit oxidative stress and ferroptosis, improving cognitive function (Chen et al., 2023a). Treatments such as propofol, ketogenic diets, mesenchymal stem cell-derived exosomes, and components like ferulate acid from traditional Chinese medicine have demonstrated potential in reducing hippocampal neuron ferroptosis and improving cognitive function by enhancing the SIRT1/Nrf2/GPX4 pathway (Wen et al., 2024; Yang et al., 2022; Liu et al., 2022; Wang et al., 2023b; Yan et al., 2024). miR-30a-5p also regulates ferroptosis by targeting SIRT1, affecting cognitive dysfunction in conditions like chronic cerebral hypoperfusion (Wang et al., 2024a). In neurodegenerative diseases such as Friedreich’s ataxia and Parkinson’s disease, SIRT1 activation helps maintain cellular iron balance, reducing oxidative stress and lipid peroxidation via Nrf2, GPX4 and FTH1, thereby protecting neurons from damage caused by ferroptosis (Lv et al., 2024; Zheng et al., 2023; Sanz-Alcázar et al., 2024). The therapeutic potential of targeting the SIRT1/Nrf2/HO-1/GPX4 pathway is further supported by research indicating that edaravone, a widely used anesthetic, mitigates depression and anxiety by inhibiting ferroptosis through this pathway (Dang et al., 2022; Shen et al., 1826). In gliomas, SIRT1-mediated ferroptosis plays a key role by finely regulating intracellular iron metabolism, redox balance, and autophagy processes. Activation of SIRT1 can enhance the antioxidant capacity of cells by deacetylating key proteins such as Nrf2 and p53, reduce the production of ROS, and inhibit lipid peroxidation, protecting nerve cells from the damage caused by ferroptosis by regulating proteins related to iron metabolism and antioxidant defense mechanisms such as Nrf2, GPX4, and FTH1. Moreover, SIRT1 activates transcription factor activating transcription factor 3 (ATF3) through its interaction with active regulator and regulation of NAD + levels, thereby inhibiting the expression of SLC7A11 and GPX4, promoting the accumulation of iron ions and lipid peroxides in cells, and aggravating ferroptosis (Chen et al., 2024c; Sun et al., 2022). Collectively, targeting SIRT1 in neuro diseases offers promising therapeutic avenues. SIRT1 activators such as resveratrol, SRT1720, and SRT2104 have been shown to alleviate neurodegenerative disease symptoms by reducing oxidative stress, enhancing autophagy flux, and promoting neuronal survival (Su et al., 2021; Zhu et al., 2022; Bai et al., 2023; Rao et al., 2024). In addition, propofol, ketogenic diet, mesenchymal stem cell-derived exosomes, and the traditional Chinese medicine were able to reduce ferroptosis in hippocampal neurons and improve cognitive function (Wen et al., 2024; Yang et al., 2022; Liu et al., 2022; Wang et al., 2023b; Yan et al., 2024). These findings underscore the multifaceted role of SIRT1 in the regulation of ferroptosis and highlight its therapeutic potential value in the treatment of neuro diseases, providing a scientific basis for the development of new therapies targeting SIRT1-mediated ferroptosis (Figure 2).

### 3.2 Liver diseases

SIRT1 is a pivotal regulator of ferroptosis in liver disease, playing a crucial role in maintaining hepatic health. In human acute liver failure tissue, SIRT1 levels are diminished, however, its activation can mitigate cell damage by modulating the ferroptosis and pyroptosis processes in hepatocytes through the regulation of the p53/GPX4/GSDMD and Nrf2/p53 signaling pathway, thereby exerting a protective effect on the liver (Zhou et al., 2024a). In a mouse model of sepsis-induced liver failure, the upregulation of SIRT1 indicates a potential role in safeguarding hepatocytes from ferroptosis, lessening liver damage, and enhancing the clinical prognosis for liver failure (Chen et al., 2022b). Furthermore, SIRT1 activators such as salidroside and ulinastatin, as well as therapeutic agents like rosa rugosa and dihydroquercetin, have been shown to significantly ameliorate liver pathological changes associated with ferroptosis by reducing oxidative stress and inflammatory responses, thus safeguarding liver cells (Wang et al., 2021; Xu et al., 2023; Lei et al., 2023; Zeng et al., 2024a). In the pathogenesis of non-alcoholic steatohepatitis (NASH), SIRT1 expression levels inversely correlate with disease progression, hinting at a significant regulatory function in ferroptosis. Activation of SIRT1 has been shown to boost the expression of antioxidant genes by deacetylating and activating Nrf2, subsequently mitigates cell damage caused by oxidative stress and dysregulated iron metabolism. SIRT1 may also curb ferroptosis in NASH by modulating other molecular pathways associated with ferroptosis, such as inhibiting lipid peroxidation and fostering iron metabolic equilibrium, offering new potential targets for NASH treatment (He et al., 2023; Yang et al., 2023). Interestingly, contrary to the hepatoprotective effects of SIRT1 activation, the loss of intestinal SIRT1 in mice shields them from ethanol-induced inflammation and liver damage by reducing liver ferroptosis. Targeting intestinal SIRT1 or alleviating ferroptosis signals in the liver may offer promising avenues for the treatment of human alcoholic liver disease (Zhou et al., 2020). Collectively, SIRT1 activation has emerged as a key therapeutic strategy. SIRT1 activators such as salidroside, ulinastatin, rosa rugosa and dihydroquercetin, have been shown to significantly improve liver pathological changes associated with ferroptosis by reducing oxidative stress and inflammatory responses (Wang et al., 2021; Xu et al., 2023; Lei et al., 2023; Zeng et al., 2024a). Interestingly, targeting intestinal SIRT1 has also been proposed as a novel approach for treating alcoholic liver disease by reducing liver ferroptosis. The findings above indicate that SIRT1-targeted therapies could serve as innovative approaches for treating various liver diseases by modulating ferroptosis pathways.

### 3.3 Lung diseases

Studies have shown that SIRT1-mediated ferroptosis plays a key role in lung injury. In acute lung injury (ALI) caused by heat attack, activation of SIRT1 has been shown to ameliorate ferroptosis in alveolar epithelial cells under heat stress. This activation alleviates the damage to the alveolar capillary barrier and maintains the barrier function of pulmonary microvascular endothelial cells, suggesting that the SIRT1/p53 axis plays a crucial role in regulating ferroptosis in ALI (Chen et al., 2022a). In lipopolysaccharide (LPS) -induced ALI, activation of SIRT1 by Meteorin-like/Meteorin-β and fibroblast growth factor (FGF) reduces ferroptosis and protects lung tissue by inhibiting p53 acetylation and Nrf2 (Chen et al., 2023b; Lin et al., 2024b). In ALI induced by sepsis, SIRT1 plays a role in inhibiting ferroptosis by activating the NADPH oxidase 4 signaling pathway, which reduces the production of ROS and the level of lipid peroxidation, and maintains the balance of iron metabolism in cells. The overexpression of growth differentiation factor 11 (GDF11) further inhibits ferroptosis by promoting the activity of SIRT1, providing a new molecular target and therapeutic strategy for treating sepsis related ALI (Wu et al., 2024). Moreover, quercetin has shown the potential to inhibit ferroptosis and alleviate ALI by activating SIRT1/Nrf2/GPX4 signaling pathway, providing a new strategy for the treatment of ALI (Deng et al., 2023). The role of SIRT1 in ALI is mainly realized by regulating oxidative stress and iron metabolism balance. By activating SIRT1, iron death can be effectively inhibited, inflammatory response and lung tissue injury can be alleviated. In addition, activation of SIRT1 by small molecule compounds, natural products, Meteorin-like/Meteorin-β, or upregulation of GDF11 may be potential strategies for the treatment of ALI. These studies provide an important scientific basis for the development of novel therapies for ALI.

### 3.4 Heart diseases

**FIGURE 3:**
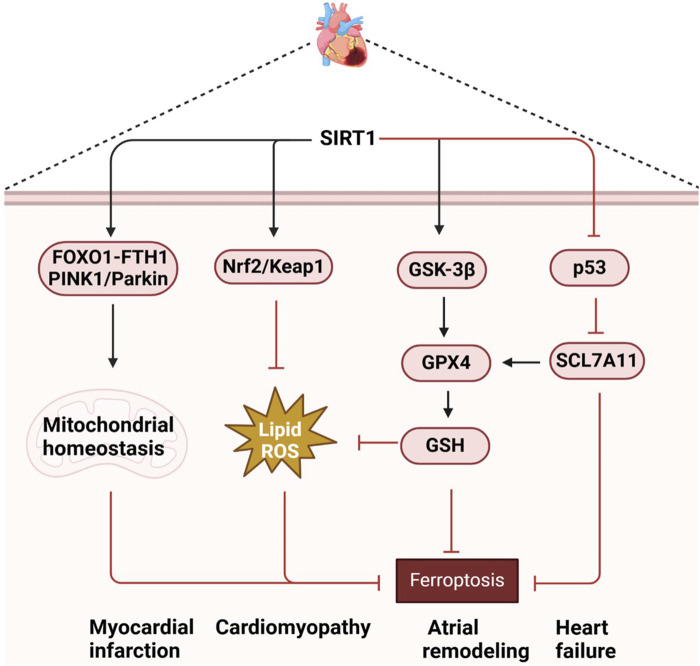
Mechanistic insights and therapeutic implications of SIRT1-mediated ferroptosis in cardiac diseases.

Bioinformatics analysis has identified SIRT1 as a key gene associated with myocardial infarction, with its role in ferroptosis being particularly significant (Jiang et al., 2022). During myocardial ischemia-reperfusion, SIRT1 activation can inhibit ferroptosis and reduce cardiac cell death, thereby protecting cardiac function. SIRT1 also interacts with Nicotinamide phosphoribosyltransferase (NAMPT) and PTEN-induced putative kinase 1 (PINK1)/Parkinson disease protein 2 (Parkin) signaling pathways to maintain mitochondrial homeostasis and promote mitochondrial autophagy, which is critical for preventing ferroptosis and myocardial damage (Ma et al., 2020; Liao et al., 2023; Ju et al., 2023). In patients with sepsis-induced cardiomyopathy (SIC), lower serum levels of GPX4 and SIRT1, along with higher levels of Creatine Kinase-Muscle/Brain (CK-MB), cardiac troponin I (cTnI), tumor necrosis factor-alpha (TNF-α), and interleukin-6 (IL-6), have been observed. Experiments showed that quercetin can reduce intracellular Fe2^+^ and prostaglandin-endoperoxide synthase 2 (PTGS2) levels, decrease the apoptosis rate, and upregulate GPX4 and ferritin levels by activating the SIRT1/p53/SLC7A11 signaling pathway. This action inhibits ferroptosis in H9C2 cells *in vitro* and alleviates SIC *in vivo* in a dose-dependent manner, suggesting a potential treatment strategy for SIC (Lin et al., 2023). In doxorubicin (DOX)-induced cardiomyopathy models, SIRT1 downregulation exacerbates ferroptosis in cardiomyocytes, while its activation reduces oxidative stress and inhibits ferroptosis by increasing the expression of antioxidant enzymes such as GPX4. Furthermore, SIRT1 enhances cellular antioxidant response through activation of Nrf2/Kelch-like ECH-associated protein 1(Keap1) signaling pathway, thus protecting cardiomyocytes from DOX-induced damage (Wang et al., 2023a; Abdel-Rahman et al., 2022; Yarmohammadi et al., 2024). SIRT1 also inhibits ferroptosis through p53-SLC7A11/GPX4 pathway, protects cardiomyocytes, and improves cardiac function in Type 1 Diabetes Mellitus (Tang et al., 2024). Icariin has been shown to protect against ethanol-induced atrial remodeling by activating the SIRT1 signaling pathway, reducing atrial ferroptosis, and inhibiting atrial fibrosis and oxidative stress. However, the protective effect of icariin is countered by the ferroptosis activator erastin and the SIRT1 inhibitor EX527 (Yu et al., 2023a). In heart failure models, SIRT1-mediated ferroptosis plays a crucial role in the pathogenesis, with AKG improving cardiac dysfunction through mitochondrial autophagy and ferroptosis inhibition mediated by the NAD + -SIRT1 signaling pathway (Yu et al., 2024). SIRT1 activation has been shown to inhibit ferroptosis in cardiomyocytes by reducing the acetylation of p53 protein, maintaining the stability of SLC7A11 protein, and increasing intracellular GSH and GPX4 levels, thereby reducing oxidative stress and lipid peroxidation (Tang et al., 2024). Natural compounds such as resveratrol and pterostilbene have shown inhibition of ferroptosis in cardiomyocytes via the SIRT1/p53 and SIRT1/Glycogen Synthase Kinase-3β (GSK-3β)/GPX4 signaling pathways, improving cardiac function and reducing cardiac remodeling in heart failure models (Zhang et al., 2023b; Zhang et al., 2024b). Natural compounds such as quercetin, icariin, resveratrol and pterostilbene reduce oxidative stress, inhibit ferroptosis of cardiomyocytes, and improve heart function by activating the SIRT1 signaling pathway (Lin et al., 2023; Yu et al., 2023a; Zhang et al., 2023b; Zhang et al., 2024b). Therefore, the regulation of SIRT1 and its associated signaling pathways not only provides insight into the molecular mechanisms of various heart diseases, but also provides potential targets for the development of new therapeutic strategies (Figure 3).

### 3.5 Kidney disease

Recent studies have highlighted the key role of SIRT1-mediated ferroptosis in multiple kidney diseases. In contrast induced nephropathy (CIN), SIRT1 activated by calorie restriction was able to reduce kidney damage via the modulation GPX4 (Fang et al., 2021). In sepsis associated acute kidney injury (SA-AKI), the exercise hormone irisin mitigates ferroptosis and kidney damage through the SIRT1/Nrf2 signaling pathway (Qiongyue et al., 2022). In Polymyxin B (PMB) -induced acute kidney injury, baicalein activated SIRT1 by reducing p53 acetylation level, thereby inhibiting ferroptosis (Yu et al., 2023b). In cisplatin-induced renal toxicity, gastrodin inhibits ferroptosis through SIRT1/FOXO3A/GPX4 signaling pathway and protects the kidney from damage (Qiu et al., 2024). The water extract of earthworms alleviates oxidative stress-induced renal cell death by enhancing SIRT1/Nrf2 signaling pathways and improving mitochondrial function (Shu et al., 2024). Baicalein, gastrodin and extract of earthworms alleviates renal cell ferroptosis induced by oxidative stress by enhancing SIRT1/Nrf2 signaling pathway and improving mitochondrial function (Yu et al., 2023b; Qiu et al., 2024; Shu et al., 2024). These researches suggest that ferroptosis can be effectively inhibited by regulating SIRT1 and its associated signaling pathways, providing a new therapeutic strategy for the treatment of kidney diseases caused by different causes.

### 3.6 Bone health

SIRT1 plays an important role in maintaining bone health by regulating ferroptosis. In disc degeneration, the SIRT1-autophagy axis may protect disc cells by inhibiting ferroptosis caused by oxidative stress (Zhou and Ruan, 2022). Moreover, in primary osteoporosis, SIRT1 is recognized as a pivotal gene linked to ferroptosis, with its expression levels potentially influencing bone metabolism and the viability of bone cells (Xia et al., 2022). Consequently, SIRT1’s role extends to Type 2 diabetic osteoporosis, where vitamin K2 promotes bone mass by activating the adenosine monophosphate-activated protein kinase (AMPK)/SIRT1 signaling pathway to suppress ferroptosis. Similarly, in postmenopausal osteoporosis, the Chinese herbal ingredient icariin is believed to have therapeutic benefits by targeting multiple ferroptosis-related pathways, including the modulation of SIRT1 (Huang et al., 2024; Wang et al., 2024b; Jin et al., 2023; Jing et al., 2019; Schluesener and Schluesener, 2014). In osteoarthritis, by deacetylating key proteins, SIRT1 activates the antioxidant response element (ARE), thereby upregulating the expression of antioxidant genes such as Nrf2, HO-1, and GPX4. This activation enhances the cellular defense against oxidative stress and suppresses the iron-mediated ROS production, which are pivotal in the pathogenesis of osteoarthritis. Consequently, the SIRT1/Nrf2 signaling axis emerges as a critical pathway in maintaining chondrocyte integrity and attenuating the degenerative processes in the joint, offering a potential therapeutic strategy for managing osteoarthritis (Zhan et al., 2023; Zhang et al., 2024c; Sun et al., 2023; Ruan et al., 2023). Together, SIRT1 activation has shown potential in maintaining bone health by inhibiting ferroptosis. Vitamin K2 and icariin have been demonstrated to promote bone mass and protect bone cells from oxidative damage by activating the AMPK/SIRT1 and SIRT1/Nrf2 pathways, respectively (Jin et al., 2023; Jing et al., 2019; Schluesener and Schluesener, 2014). These findings highlight SIRT1’s central role in regulating ferroptosis, protecting bone cells from oxidative damage, and maintaining bone health.

### 3.7 Cancer

SIRT1-mediated ferroptosis is increasingly recognized as a pivotal factor in diverse cancer treatment strategies. Bioinformatics analysis has revealed a significant association between SIRT1 and ferroptosis in both hepatocellular carcinoma (HCC), gastric cancer and Ewing’s sarcoma, highlighting its potential as a therapeutic target in these diseases (Sui et al., 2019; Niu et al., 2023; Jiao et al., 2023). In HCC, SIRT1 inhibition by protocadherin 20 promotes ferroptosis via reducing the expression of SLC7A11, GPX4 and GSH, while increasing MDA, ROS and intracellular iron levels, resulting in a significant decrease in cell viability, colony-forming ability, and the growth and size of tumor (Jun et al., 2023). In the context of gastric cancer, SIRT1 collaborates with APE1 to stimulate cancer cell ferroptosis by repressing p53, thereby curbing cancer cell proliferation (Zhao et al., 2023a). Furthermore, the loss of long non-coding RNA DACT3-AS1 in exosomes derived from cancer-associated fibroblasts (CAFs) promotes gastric tumor malignant transformation and oxaliplatin resistance by affecting the miR-181a-5p/SIRT1 axis, a process involved in the regulation of ferroptosis (Qu et al., 2023). In addition, in colorectal cancer, ropivacaine enhances cisplatin sensitivity by inhibiting SIRT1 expression, an effect achieved in part by promoting ferroptosis (Zeng et al., 2024b). In melanoma studies, ubiquitin specific peptidase 22 controls melanoma metastasis and sensitivity to ferroptosis through the SIRT1/phosphatase and tensin homolog deleted on chromosome 10 (PTEN)/phosphoinositol-3 kinase (PI3K) signaling pathway, suggesting that activation of the SIRT1 pathway may enhance melanoma cells’ sensitivity to ferroptosis (Sun et al., 2024). In the study of lung adenocarcinoma, high doses of β-nicotinamide mononucleotide promote ferroptosis and inhibit lung cancer cell growth through the excess nicotinamide-mediated SIRT1/AMPK/acetyl-coA carboxylase (ACC) signaling pathway (Zhang et al., 2023a). In head and neck cancer, the activation of SIRT1 facilitates the epithelial-mesenchymal transition (EMT), thereby enhancing cancer cells’ susceptibility to ferroptosis. Conversely, the inhibition of SIRT1 diminishes ferroptosis. Additionally, SIRT1 plays a role in governing ferroptosis by modulating the expression of GPX4, SLC7A11, and SLC3A2 (Lee et al., 2020). In paclitaxel-tolerant persister head and neck cancer (HNC) cell lines, SIRT1 activation promotes ferroptosis by increasing mitochondrial fatty acid oxidation via facilitating the dispersion and localization of lipid droplets on mitochondria (You et al., 2021). In addition, in studies of chronic lymphocytic leukemia (CLL), activation or inhibition of SIRT1 influenced the sensitivity of CLL cells to ferroptosis, suggesting a potential role for SIRT1 in regulating ferroptosis in CLL cells (Pan et al., 2022). These results underscore the potential of SIRT1 modulators (activators or inhibitors) and the ferroptosis pathway it regulates, as a novel therapeutic strategy for cancer treatment. Future research will further explore the specific mechanisms of action of SIRT1 modulators in different types of cancer, providing a scientific basis for the development of more effective cancer therapies.

### 3.8 Inflammatory disease

SIRT1-mediated ferroptosis plays an important role in the pathological process of mastitis. The activation of SIRT1 can inhibit the activation of inflammasome and the release of inflammatory cytokines, and reduce the inflammatory response. At the same time, SIRT1 deacetylates multiple transcription factors, such as Nrf2, promotes its entry into the nucleus and activates the expression of antioxidant stress genes, such as HO-1 and GPX4, the activation of which helps mitigate cell damage caused by ferroptosis. In mastitis, SIRT1 activation helps to reduce intracellular iron content and inhibit lipid peroxidation, thereby reducing ferroptosis and protecting breast tissue from damage (Zhou et al., 2024b; Zhao et al., 2023b; Zhao et al., 2023c). Natural compounds and small-molecule activators that enhance SIRT1 activity could be developed as novel therapeutics for mitigating inflammatory responses and protecting tissue from ferroptosis-induced damage.

### 3.9 Diabetes

In diabetes mellitus, SIRT1-mediated ferroptosis plays an important role in islet β-cell dysfunction. Research has shown that hyperglycemia inhibits the expression of SIRT1 in islet β-cells, leading to decreased levels of the antioxidant enzyme GPX4 and increased expression of the ferroptosis-related protein TFR1. This weakens the cells’ antioxidant capacity, making them more susceptible to oxidative stress and ultimately resulting in ferroptosis. Stabilizing SIRT1 activity alleviates ferroptosis in islet β-cells, improves insulin secretion, and mitigates hyperglycemia symptoms (Zhang et al., 2022). SIRT1 is also involved in the development of diabetic complications through its regulation of ferroptosis. Under high-glucose conditions, SIRT1 activity is reduced, leading to increased ferroptosis. Activating SIRT1 can inhibit ferroptosis and reduce pathological damage in diabetic complications. For example, in diabetic retinopathy (DR), SIRT1 suppresses inflammation and retinal vascular damage by regulating HMGB1 deacetylation and inhibiting ferroptosis (Peng et al., 2025). Inhibition of SIRT1 reduces Nrf2 activity, decreases the expression of antioxidant-related molecules, and exacerbates ferroptosis. Astragaloside-IV can enhance SIRT1 and Nrf2 activity, boost cellular antioxidant capacity, reduce hyperglycemia-induced ferroptosis, and protect retinal pigment epithelial (RPE) cells from damage, offering a potential therapeutic strategy for DR (Tang et al., 2022). Flavanones can increase SIRT1 activity, inhibit ferroptosis through the FOXO3a and Nrf2 signaling pathways, and alleviate renal tubular epithelial cell injury induced by high glucose (Zhou et al., 2025). Additionally, activating the SIRT1/Nrf2/p62 pathway can promote the healing of diabetic foot ulcers, possibly mediated by autophagy-dependent ferroptosis (Han et al., 2024). In diabetic peripheral neuropathy (DPN), inactivation of SIRT1 promotes the production of mitochondrial ROS, leading to dysfunction and ferroptosis in Schwann cells. Activation of the AMPK/SIRT1/PGC-1α signaling pathway by honokiol alleviates hyperglycemia-induced oxidative stress and ferroptosis, thereby improving cell function (Liang et al., 2023; Hu et al., 2023). These findings suggest that SIRT1 and its regulation of ferroptosis are crucial in diabetic complications and may represent a novel therapeutic target for these conditions.

Together, the diverse roles of SIRT1 across multiple disease types underscore its significance as a promising therapeutic target. A variety of therapeutic agents have been explored for modulating SIRT1 in ferroptosis-related diseases. Table 1 summarizes key SIRT1-targeted interventions, their mechanisms, and potential clinical applications across various pathological conditions. This consolidated information provides a reference for ongoing research and potential translational applications in ferroptosis-associated diseases (Table 1).

**TABLE 1:** Therapeutic strategies targeting SIRT1 in ferroptosis-associated diseases.

Disease type	SIRT1 regulation	Therapeutic strategies	Mechanism	Clinical potential	Ref
Neurological Diseases	Activation	Resveratrol, SRT1720, SRT2104, Propofol, Ketogenic diet, Ferulic acid	Enhance Nrf2/GPX4, reduces oxidative stress, mitigates lipid peroxidation	Protect neurons from ferroptosis in neurodegenerative disorders and cognitive decline	Wen et al. (2024), Yang et al. (2022), Liu et al. (2022), Wang et al. (2023b), and Yan et al. (2024)
Liver Diseases	Activation	Salidroside, Ulinastatin, Rosa rugosa, Dihydroquercetin	Regulate p53/GPX4/GSDMD and Nrf2/p53, decreases oxidative stress	Protect against liver failure, sepsis, and NASH	Wang et al. (2021), Xu et al. (2023), Lei et al. (2023), and Zeng et al. (2024a)
Lung Diseases	Activation	Meteorin-like, Meteorin-β, FGF, GDF11, Quercetin	Suppress p53 acetylation, activate SIRT1/Nrf2/GPX4, maintains iron homeostasis	Alleviate ALI	Chen et al. (2022a), Chen et al. (2023b), Lin et al. (2024b), Wu et al. (2024), and Deng et al. (2023)
Cardiovascular Diseases	Activation	Quercetin, Resveratrol, Icariin, Pterostilbene,	Activate Nrf2/Keap1, SIRT1/p53/SLC7A11, inhibit ferroptosis in cardiomyocytes	Protect against myocardial infarction, ischemia-reperfusion injury, and doxorubicin-induced cardiotoxicity	Lin et al. (2023), Wang et al. (2023a), Abdel-Rahman et al. (2022), Yarmohammadi et al. (2024), Tang et al. (2024), Yu et al. (2023a), Yu et al. (2024), Zhang et al. (2023b), and Zhang et al. (2024b)
Kidney Diseases	Activation	Baicalein, Gastrodin, Earthworm extract	Enhance SIRT1/Nrf2, reduce oxidative stress	Mitigates acute kidney injury	Yu et al. (2023b), Qiu et al. (2024), and Shu et al. (2024)
Bone Health	Activation	Vitamin K2, Icariin	Activate AMPK/SIRT1, inhibit oxidative stress-induced ferroptosis	Prevent osteoporosis and osteoarthritis	Jin et al. (2023), Jing et al. (2019), and Schluesener and Schluesener (2014)
Cancer	Activation or Inhibition	Protocadherin 20, Ropivacaine, β-nicotinamide mononucleotide	Regulate SIRT1/AMPK, PTEN/PI3K, modulate ferroptosis sensitivity in tumors	Enhances ferroptosis-based strategies in HCC, colorectal cancer, melanoma, and lung adenocarcinoma	Jun et al. (2023), Zeng et al. (2024b), Sun et al. (2024), and Zhang et al. (2023a)
Mastitis	Activation	Schisandrin B, saikosaponin A, diosmetin	Regulate SIRT1/p53/SLC7A11, Nrf2, GPX4	Alleviate mastitis	Zhou et al. (2024b), Zhao et al. (2023b), and Zhao et al. (2023c)
Diabetes	Activation	Astragaloside-IV, Flavanones, Honokiol	Enhance FOXO3a/NRF2, preserve β-cell function	Ameliorate diabetes and its complications	Tang et al. (2022), Zhou et al. (2025), Han et al. (2024), Liang et al. (2023), and Hu et al. (2023)

## 4 Future research directions and prospects

The role of SIRT1 in the regulation of ferroptosis has emerged as a promising area of research with significant therapeutic potential in a multiple of diseases. While significant progress has been made in understanding the role of SIRT1 in ferroptosis, there are still many challenges to overcome. The integration of new technologies and the pursuit of innovative research directions will be instrumental in advancing the field and unlocking the therapeutic potential of targeting SIRT1 and ferroptosis. Future research should focus on elucidating the precise molecular mechanisms by which SIRT1 regulates ferroptosis. This includes investigating the full spectrum of SIRT1 targets, the epigenetic changes it induces, and how these contribute to the susceptibility of target cells to ferroptosis. Research could focus on identifying SIRT1-dependent gene regulatory networks that modulate ferroptosis. Investigating the interplay between SIRT1 and other cellular pathways, such as autophagy and the unfolded protein response, will also be crucial for a comprehensive understanding of ferroptosis regulation. What’s more, the development of small molecules that specifically target SIRT1 or its regulatory pathway is a promising avenue. This includes the design of more potent and selective SIRT1 modulators and the evaluation of their efficacy in preclinical models of diseases characterized by ferroptosis, such as kidney diseases, neurodegenerative disorders, and cancer. Moreover, the development of predictive biomarkers for ferroptosis sensitivity is crucial. Future studies should aim to identify and validate biomarkers that can predict a patient’s response to ferroptosis-inducing therapies, allowing for personalized treatment strategies. Translational research efforts should be directed towards the design of clinical trials assessing the safety and efficacy of SIRT1-targeted ferroptosis induction in patients. Notably, long-term studies are needed to assess the safety and efficacy of SIRT1 modulation in inducing ferroptosis.

Through the diligent exploration of these research avenues, the scientific community can potentially unlock the therapeutic potential of SIRT1 and ferroptosis as novel strategies in human disease. This advancement may provide renewed hope for patients who have exhausted conventional treatment options and are in dire need of innovative therapeutic interventions.

## 5 Discussion

Our review underscores the multifaceted role of SIRT1 in modulating ferroptosis, highlighting its potential as a therapeutic target. The intricate interplay between SIRT1 and the molecular machinery governing ferroptosis offers a rich avenue for therapeutic intervention. SIRT1’s ability to deacetylate and thereby activate key proteins involved in ferroptosis, such as GPX4 and FOXO3A, positions it as a nodal point in the regulation of this cell death process. By maintaining cellular redox balance and influencing iron homeostasis, SIRT1 contributes to the cellular resistance against ferroptosis. The therapeutic potential of SIRT1 in modulating ferroptosis is vast and spans a range of diseases, including neurodegenerative disorders, cancer, and kidney diseases. The activation of SIRT1 has been shown to ameliorate ferroptosis-associated cell death in various models, suggesting its utility in developing protective strategies against diseases where ferroptosis plays a pathogenic role. However, the field faces challenges, including the need for a deeper understanding of the molecular underpinnings of SIRT1’s role in ferroptosis and the development of targeted therapies that can effectively harness this enzyme’s activity. In conclusion, the regulation of ferroptosis by SIRT1 represents a burgeoning frontier in cellular biology with significant therapeutic implications. As we continue to unravel the complexities of this process, we edge closer to a future where the modulation of ferroptosis through SIRT1 activation may offer novel treatment strategies for a host of diseases. Our review establish a foundation for future research and pave the way for novel therapeutic strategies that could harness the potential of ferroptosis in biological and medical contexts.
